# Impact of an Educational Training Program on the Knowledge, Attitude, and Perceived Barriers of Community Pharmacists Towards Obesity and Overweight Management in Malaysia

**DOI:** 10.3389/fpubh.2021.720939

**Published:** 2021-09-01

**Authors:** Rohit Kumar Verma, Wei Wen Chong, Nur Akmar Taha, Thomas Paraidathathu

**Affiliations:** ^1^Department of Pharmacy Practice, School of Pharmacy, International Medical University, Kuala Lumpur, Malaysia; ^2^Centre of Quality Management of Medicines, Faculty of Pharmacy, Universiti Kebangsaan Malaysia, Kuala Lumpur, Malaysia; ^3^Faculty of Pharmacy, Cyberjaya University College of Medical Sciences, Cyberjaya, Malaysia; ^4^Faculty of Health and Medical Sciences, Taylor's University, Subang Jaya, Malaysia

**Keywords:** weight management, community pharmacist, community pharmacy, obesity, overweight, educational training

## Abstract

**Objective:** To evaluate the impact of an educational training program on the knowledge, attitude and perceived barriers of community pharmacists (CPs) towards obesity and overweight management.

**Methods:** This interventional study, which consisted of an educational training program, was conducted on a single cohort of Malaysian CPs. Thirty CPs attended the educational training program. The educational training program was delivered through didactic lectures, case studies and small group discussions, and consisted of various sessions covering different topics related to weight management. A validated questionnaire was used to assess the impact of the intervention on the CPs' knowledge, attitude, and perceived barriers.

**Results:** The overall mean knowledge score increased both immediately after (14.93 ± 1.62) and 30 days following the intervention (17.04 ± 2.51), and the increment was statistically significant 30 days following the intervention (*p* = 0.001) compared to both pre-intervention and immediate-post intervention stages. After the intervention, the participants had a more positive attitude towards the provision of weight management service (WMS) in community pharmacies. They had significantly stronger perceptions about the importance of their role to manage overweight and obesity and their professional competence to treat obese patients. In addition, the barrier of not having space in pharmacy to perform proper counselling for weight management and the barrier of not having training sessions in the area of obesity management were perceived to be significantly less important post-intervention.

**Conclusion:** This study showed the potential positive impact of an educational training program on CPs knowledge, attitudes and perceived barriers towards WMS.

## Introduction

Community pharmacy-based weight management service (WMS) is a potential key area through which community pharmacists (CPs) can contribute to the public health agenda, especially amid the background of increasing prevalence of obesity in populations around the world and its burden on society. A systematic review of 10 studies worldwide had demonstrated the effectiveness of pharmacy-based weight management interventions in producing positive outcomes, including clinically significant weight loss (1).

CPs providing WMS would be expected to be well-versed with knowledge regarding various aspects of evidence-based obesity management such as dietary approaches, physical activity recommendations, and pharmacological therapies, as well as associated health risks. However, a lack of knowledge and training related to obesity management or weight reduction counseling have been reported as barriers to pharmacist-led interventions. This was exemplified in a mail survey of Texan CPs where they were only somewhat comfortable with counseling patients on the various aspects of the management of obesity and were neutral in terms of their confidence to achieve positive outcomes as a result of their counseling activities (2). This perception was related to their years in practice in which the longer they were in practice, the more confident they were in achieving positive outcomes without the use of medication (2). In addition, semi-structured interviews with Australian CPs highlighted the lack of proper training in weight management-related issues in terms of dietary approaches, physical activity recommendations, or behavioral therapies (3). Another Australian study that utilized case vignettes to evaluate CPs' weight management recommendations commented that some of their recommendations were not evidence-based (4).

CPs have also expressed their willingness to participate in training and accreditation in order to obtain wider acknowledgment of their professional expertise in WMS (3). In fact, a study that reported perceived lack of knowledge as a barrier also expected an improvement in this perception as well as self-confidence with proper training (2). Perceived training needs in weight management identified by CPs include various aspects such as diet, exercise, behavioral therapy, obesity-related comorbidities, point-of-care testing (measurement of cholesterol, estimation of body fat), consultation skills, and counseling on weight-loss drugs (5).

Educational opportunities in weight management should be offered for CPs, including continuing pharmaceutical education programs and seminars that provide focused training on disease state management strategies in obesity and the administration and interpretation of point-of-care testing and physical assessment. This study aimed to evaluate the impact of an educational training program on the knowledge, attitude, and perceived barriers of CPs toward obesity and overweight management.

## Methods

### Study Design

This interventional study, which consisted of an educational training program, was conducted on a single cohort of Malaysian CPs. The invitation to participate in this educational training program was extended through the Malaysian Pharmacists Society (MPS) to all CPs who were currently practicing in Klang Valley, Malaysia. Thirty CPs attended the educational training program.

The one-day educational training program on 18 November 2017 at the MPS headquarters in Puchong, Selangor, lasted for 8 hours. The educational training program was delivered by several experts in weight management, consisting of pharmacists, a nutritionist, and a dietitian. The content and materials for the educational training program were designed based on a training manual for pharmacists for management of obesity and overweight. This manual was developed through collaboration between the Malaysian Academy of Pharmacy and the MPS for a community service program (MyWeight MyHealth). The educational training program was delivered through didactic lectures, case studies and small group discussions, and consisted of various sessions covering different topics related to weight management as follows: (i) overview of overweight and obesity (ii) developing an individualized care plan (iii) dietary approaches and exercises in weight management (iv) behavioral modifications in weight management (v) pharmacotherapy in weight management and (vi) roles of CPs in the management of overweight and obese patients.

### Questionnaire Development and Dissemination

A questionnaire was developed for the purpose of this study. Content validation of the questionnaire was done by experts (pharmacists, nutritionists, dietitians). The questionnaire comprised of several sections including (1) sociodemographic and practice characteristics of CPs, (2) knowledge of CPs about obesity and overweight, diet, nutrition, behavioral aspects of obesity and its management, and pharmacotherapy of overweight and obesity, (3) attitudes of CPs towards weight management and weight management training programs, and (4) perceived barriers of CPs regarding weight management.

The knowledge section of the questionnaire was divided into four subsections and consisted of 20 items, for which participants selected either “true” or “false” for each item. Each correct response on the knowledge section was scored ‘1’, while each incorrect response was scored ‘0’. The mean score for each knowledge subsection and overall mean knowledge score were calculated for all CPs. On the other hand, responses for the attitudes and barriers sections were recorded using a five-point Likert scale, ranging from 1 = strongly disagree to 5 = strongly agree.

The questionnaire was distributed to all CPs who attended the educational training program, and they were requested to complete it at the start of the program. At the end of the educational training program, the CPs were requested to fill the same questionnaire again to assess the immediate impact of the educational training program on their knowledge, attitudes, and perceived barriers. After 30 days, the same questionnaire was mailed to the participants again to assess knowledge retention, and changes in attitudes and perceived barriers. The participation of CPs in this study was voluntary with no compensation paid. Written consent was obtained from the participants before dissemination of the questionnaire.

### Statistical Analysis

Statistical analysis was performed using SPSS version 18. Descriptive statistics were used to describe the demographic characteristics of the participants. Wilcoxon's Signed-Rank Tests were used to evaluate the impact of interventions, wherever applicable. The significance level for all statistical analyses was set as *P* < 0.05.

## Results

### Sociodemographic and Practice Characteristics

Table 1 displays the demographic and practice characteristics of the 30 participants. Female participants (*n* = 19, 63.3%) were almost twice the number of male participants (*n* = 11, 36.7%). Those in the age group of 25–34 years constituted the highest number of participants (*n* = 11, 36.7%). In terms of educational level, more than three-quarters of the participants had a Bachelor of Pharmacy degree (*n* = 23, 76.7%) as their highest qualification. Nearly half of the participants had between 1–5 years of experience as a community pharmacist (*n* = 14, 46.7%), and an equal number of participants (*n* = 13, 43.3%) worked in chain pharmacies and independent pharmacies, respectively. Only five participants (16.7%) worked in a pharmacy with a proper weight management program, while 24 participants (80.0%) attended training on weight management for the first time. Twenty (66.7%) participants had at least two patients visiting their pharmacy in a month to obtain treatment or counseling regarding weight issues.

**Table 1:** Sociodemographic and practice characteristics of community pharmacists (*N* = 30).

**Characteristics**		**Frequency (*n*)**	**Percentage (%)**
Age group (years)	18–24	6	20.0
	25–34	11	36.7
	35–44	3	10.0
	45–54	1	3.3
	55–64	6	20.0
	65 and above	3	10.0
Gender	Male	11	36.7
	Female	19	63.3
Ethnicity	Malay	9	30.0
	Chinese	18	60.0
	Indian	3	10.0
Highest education level	Bachelor of Pharmacy degree	23	76.7
	Master's degree	5	16.7
	Doctorate degree	1	3.3
	Other	1	3.3
Years of experience as a community pharmacist (in years)	<1	5	16.7
	1–5	14	46.7
	6–10	4	13.3
	More than 10	7	23.3
Participants working in a pharmacy that had a proper weight management programme		5	16.7
Participants attending training on weight management for the first time		24	80.0
On an average of a month, number of patients visiting the pharmacy to get treatment/counseling regarding weight management issues	0–1	10	33.3
	2–5	8	26.7
	5–10	8	26.7
	10–15	4	13.3
Type of community pharmacy	Independent pharmacy	13	43.3
	Chain pharmacy	13	43.3
	Other	4	13.3

### Impact of the Educational Training Program on Knowledge of Weight Management

Table 2 shows the knowledge scores at pre-, immediate-post-, and 30-days post-intervention stages of the participants. Before the intervention, the overall mean knowledge score was 14.50 ± 2.45. The overall mean knowledge score increased both immediately after (14.93 ± 1.62) and 30 days following the intervention (17.04 ± 2.51). The increment was statistically significant 30 days following the intervention (*P* = 0.001) compared to both pre-intervention and immediate-post intervention stages. The knowledge score for each section increased both immediately after and 30 days following the intervention, except that there was a non-significant decline in knowledge score in the section of “Diet, Nutrition, and Physical Activity” immediately after intervention (from 3.07 ± 2.16 to 2.76 ± 1.17; *P* = 0.657). Besides, statistically significant increments of knowledge scores for all sections of the knowledge test were also achieved 30 days following the intervention compared to both pre-intervention and immediate-post-intervention stages.

**Table 2:** Knowledge score of participants.

**Sections**		**Pre-intervention scores^**f**^Mean (SD)**	**Immediate post-intervention scores^**f**^ Mean (SD)**	***P* value^**g**^**	**Pre-intervention scores^**f**^ Mean (SD)**	**30-day post-intervention scores^**f**^ Mean (SD)**	***P* value^**g**^**	**Immediate post-intervention scores^**f**^ Mean (SD)**	**30-day post-intervention scores^**f**^ Mean (SD)**	***P* value^**g**^**
Section 1^a^	Overview of Overweight and Obesity	6.40 (0.81)	6.53 (0.63)	0.973	6.40 (0.81)	6.80 (0.50)	**0.040**	6.53 (0.63)	6.80 (0.50)	**0.039**
Section 2^b^	Diet, Nutrition, and Physical Activity	3.07 (2.16)	2.76 (1.17)	0.657	3.07 (2.16)	3.40 (0.91)	**0.018**	2.76 (1.17)	3.40 (0.91)	**0.047**
Section 3^c^	Behavioral Intervention	2.20 (0.55)	2.76 (0.86)	**0.003**	2.20 (0.55)	3.36 (0.95)	**0.001**	2.76 (0.86)	3.36 (0.95)	**0.009**
Section 4^d^	Pharmacotherapy	2.83 (0.75)	2.87 (0.68)	0.843	2.83 (0.75)	3.48 (0.71)	**0.007**	2.87 (0.68)	3.48 (0.71)	**0.014**
Overall^e^		14.50 (2.45)	14.93 (1.62)	0.233	14.50 (2.45)	17.04 (2.51)	**0.001**	14.93 (1.62)	17.04 (2.51)	**0.001**

*SD, standard deviation. Possible score range:*

a *Section 1 = 0–7*,

b *Section 2 = 0–4*,

c *Section 3 = 0–5*,

d *Section 4 = 0–4*,

e *Overall=0–20*,

f *Higher score represents higher knowledge*,

g *P values are based on Wilcoxon's Signed-Rank Test*.

*Note: The bold values in the tables indicate statistical significance at p < 0.05*.

### Impact of the Educational Training Program on Attitude Towards Weight Management Training and Weight Management Service

Table 3 shows participants' attitudes towards weight management training and WMS before, immediately after, and 30 days after the intervention. Regarding participants' attitudes towards weight management training, there was a significantly stronger level of agreement that only certified pharmacists who have attended such training should be eligible to offer WMS (*P* = 0.007, pre- vs. 30-day post-intervention).

**Table 3:** The impact of the intervention on attitude of participants towards weight management training and weight management services.

**Item Description**	**Pre-intervention Mean (SD)^**a**^**	**Immediate post-interventionMean (SD)^**a**^**	***P* value^**b**^**	**Pre-interventionMean (SD)^**a**^**	**30-day post-intervention Mean (SD)^**a**^**	***P* value^**b**^**	**Immediate post-intervention Mean (SD)^**a**^**	**30-day post-interventionMean (SD)^**a**^**	***P* value^**b**^**
I think I need training on weight management.	4.50 (0.51)	4.37 (0.49)	**0.046**	4.50 (0.51)	4.52 (0.59)	0.746	4.37 (0.49)	4.52 (0.59)	0.288
I think weight management training will improve my confidence while managing patients who are overweight/obese.	4.57 (0.50)	4.40 (0.50)	0.096	4.57 (0.50)	4.56 (0.51)	0.892	4.40 (0.50)	4.56 (0.51)	0.288
I think professionals from other professions such as nutritionists and dieticians should also be a training provider.	4.47 (0.7)	4.40 (0.56)	0.666	4.47 (0.7)	4.64 (0.57)	0.320	4.40 (0.56)	4.64 (0.57)	0.132
I think weight management training should be provided by the Ministry of Health in conjunction with Malaysian Pharmaceutical Society and pharmaceutical companies dealing with anti-obesity drugs/supplements.	4.10 (0.85)	4.03 (0.96)	0.714	4.10 (0.85)	4.36 (0.86)	0.172	4.03 (0.96)	4.36 (0.86)	0.119
I think only certified pharmacists who have attended weight management training should be eligible to offer weight management services.	3.33 (0.88)	3.33 (0.96)	0.927	3.33 (0.88)	4.00 (1.11)	**0.007**	3.33 (0.96)	4.00 (1.11)	**0.014**
I think minor weight loss can produce good clinical outcome for overweight and obese patients.	4.07 (0.64)	4.23 (0.50)	0.166	4.07 (0.64)	4.52 (0.59)	**0.012**	4.23 (0.50)	4.52 (0.59)	0.077
I think I have an important role to manage overweight and obesity.	4.27 (0.52)	4.37 (0.49)	0.257	4.27 (0.52)	4.60 (0.58)	**0.037**	4.37 (0.49)	4.60 (0.58)	0.169
I think I am professionally competent to treat patients with BMI more than 30 kg/m^2^.	3.13 (0.97)	3.63 (0.81)	**0.013**	3.13 (0.97)	4.04 (1.14)	**0.001**	3.63 (0.81)	4.04 (1.14)	**0.047**
I think as a pharmacist I should refer overweight/obese patients to other professionals rather than to attempt to treat them.	2.83 (0.91)	2.73 (1.01)	0.670	2.83 (0.91)	2.24 (0.83)	**0.004**	2.73 (1.01)	2.24 (0.83)	**0.040**
I think I should only counsel patients who are overweight and obese when the patient requests it.	2.77 (0.97)	2.60 (1.00)	0.379	2.77 (0.97)	1.84 (0.90)	**0.001**	2.60 (1.00)	1.84 (0.90)	**0.002**
I think I should not provide any assistance to overweight /obese patients if I am not reimbursed for the service.	2.47 (0.86)	2.17 (0.70)	0.118	2.47 (0.86)	1.96 (0.45)	**0.001**	2.17 (0.70)	1.96 (0.45)	0.068
I think medications for weight management should only be offered when other risk factors such as diabetes mellitus or hypertension are present.	2.77 (1.10)	2.73 (1.01)	0.789	2.77 (1.10)	2.40 (0.82)	0.056	2.73 (1.01)	2.40 (0.82)	0.081
I think adults with BMI above 25kg/m^2^ should be offered anti-obesity drugs.	2.53 (0.82)	2.33 (0.88)	0.318	2.53 (0.82)	2.28 (0.89)	0.289	2.33 (0.88)	2.28 (0.89)	0.763
I think adults with BMI above 30 kg/m2 should be offered anti-obesity drugs.	3.47 (0.94)	3.97 (0.89)	**0.007**	3.47 (0.94)	4.48 (0.82)	**0.001**	3.97 (0.89)	4.48 (0.82)	**0.032**
I think the following services/practices are important for me to provide in my pharmacy:									
a) Obesity screening services	4.10 (0.66)	4.30 (0.53)	0.124	4.10 (0.66)	4.52 (0.59)	**0.016**	4.30 (0.53)	4.52 (0.59)	0.155
b) Counseling about dietary habits	4.30 (0.47)	4.23 (0.50)	0.782	4.30 (0.47)	4.56 (0.58)	0.087	4.23 (0.50)	4.56 (0.58)	**0.033**
c) Counseling about physical activity	4.27 (0.52)	4.27 (0.45)	0.796	4.27 (0.52)	4.56 (0.58)	0.057	4.27 (0.45)	4.56 (0.58)	0.051
e) Provide anti-obesity drugs from pharmacy	3.57 (1.01)	3.87 (0.90)	0.139	3.57 (1.01)	4.40 (0.77)	**0.001**	3.87 (0.90)	4.40 (0.77)	**0.017**
I do not prefer to advise anything to overweight/obese patients on lifestyle/dietary modifications as I know these methods are not effective in weight management.	2.20 (1.06)	2.10 (0.92)	0.736	2.20 (1.06)	1.40 (0.58)	**0.001**	2.10 (0.92)	1.40 (0.58)	**0.001**
I would like to give anti-obesity medications only to achieve weight loss instead of advises on lifestyle/dietary modifications.	1.90 (0.89)	1.87 (0.82)	0.870	1.90 (0.89)	1.44 (0.58)	**0.019**	1.87 (0.82)	1.44 (0.58)	0.100

*SD, standard deviation; BMI, body mass index*.

a *Mean reflects the score on a 5-point Likert Scale (1 = strongly disagree; 5 = strongly agree)*.

b *P values are based on Wilcoxon's Signed-Rank Test*.

*Note: The bold values in the tables indicate statistical significance at p < 0.05*.

Participants had a significantly stronger level of agreement on the importance of their role to manage overweight and obesity following the intervention (*P* = 0.037, pre- vs. 30-day post-intervention). In addition, participants also had a significantly stronger level of agreement, both immediately and 30-day post-intervention, that they are professionally competent to treat obese patients (*P* = 0.013 and *P* = 0.001 respectively). There was a significantly stronger level of disagreement on the following statements “I think as a pharmacist I should refer overweight/obese patients to other professionals rather than to attempt to treat them” (*P* = 0.004, pre- vs. 30-day post-intervention) and “I think I should only counsel patients who are overweight and obese when the patient requests it” (*P* = 0.001, pre- vs. 30-day post-intervention).

Following the intervention, participants also had stronger agreement on the importance of providing WMS in their pharmacy, such as obesity screening services and provision of anti-obesity drugs (*P* = 0.016 and *P* = 0.001 respectively, pre-vs. 30-day post-intervention). Participants also reported a stronger level of disagreement that they do not prefer to advise anything related to lifestyle or dietary modifications (*P* = 0.001, pre-vs. 30-day post-intervention), and would give anti-obesity medications only to achieve weight loss (*P* = 0.019, pre- vs. 30-day post-intervention).

### Impact of the Educational Training Program on Perceived Barriers Towards Weight Management Service

Perceived barriers of participants towards WMS were assessed before, immediately after, and 30 days after the intervention (Table 4). Following the intervention, there was a significantly stronger level of agreement with barriers related to the lack of manpower and reluctance of patients to pay pharmacists (*P* = 0.001 and *P* = 0.004 respectively, pre- vs. 30-day post-intervention).

**Table 4:** The impact of the intervention on perceived barriers of participants towards weight management services.

**Item Description**	**Pre-intervention Mean (SD)^**a**^**	**Immediate post-intervention Mean (SD)^**a**^**	***P* value^**b**^**	**Pre-intervention Mean (SD)^**a**^**	**30-day post-intervention Mean (SD)^**a**^**	***P* value^**b**^**	**Immediate post-intervention Mean (SD)^**a**^**	**30-day post-intervention Mean (SD)^**a**^**	***P* value^**b**^**
Lack of time	2.57 (0.90)	2.50 (0.82)	0.554	2.57 (0.90)	2.38 (0.65)	0.288	2.50 (0.82)	2.38 (0.65)	0.337
Lack of manpower	2.70 (0.95)	2.70 (1.05)	0.859	2.70 (0.95)	3.54 (0.78)	**0.001**	2.70 (1.05)	3.54 (0.78)	**0.001**
Lack of weight management education material	3.37 (1.00)	3.57 (1.04)	0.305	3.37 (1.00)	2.46 (0.83)	**0.001**	3.57 (1.04)	2.46 (0.83)	**0.001**
Lack of useful clinical practice guideline for obesity	2.77 (0.86)	3.07 (0.94)	0.177	2.77 (0.86)	2.29 (0.46)	**0.018**	3.07 (0.94)	2.29 (0.46)	**0.001**
Lack of proper referral system for overweight/obese patients	3.53 (0.97)	3.50 (1.04)	0.644	3.53 (0.97)	3.75 (0.53)	0.234	3.50 (1.04)	3.75 (0.53)	0.256
Lack of training sessions in the management of overweight/obesity	3.30 (1.02)	2.93 (1.17)	0.073	3.30 (1.02)	2.33 (0.70)	**0.001**	2.93 (1.17)	2.33 (0.70)	**0.012**
Lack of space for proper counseling	3.03 (1.07)	2.97 (1.19)	0.939	3.03 (1.07)	2.37 (0.64)	**0.003**	2.97 (1.19)	2.37 (0.64)	**0.014**
Lack of awareness among overweight/obese patients	3.57 (0.97)	3.67 (0.80)	0.591	3.57 (0.97)	3.75 (0.53)	0.402	3.67 (0.80)	3.75 (0.53)	0.839
Reluctance among overweight/obese patients to obtain advice from community pharmacists	2.67 (0.99)	2.93 (0.98)	0.340	2.67 (0.99)	2.21 (0.42)	**0.030**	2.93 (0.98)	2.21 (0.42)	**0.001**
Reluctance among overweight/obese patients to pay pharmacists for weight management services	3.77 (0.77)	3.70 (0.99)	0.924	3.77 (0.77)	4.33 (0.82)	**0.004**	3.70 (0.99)	4.33 (0.82)	**0.015**
Preference among overweight/obese patients to purchase anti-obesity medications instead of getting advice on lifestyle/dietary modification	3.23 (1.04)	3.33 (0.92)	0.593	3.23 (1.04)	2.33 (0.57)	**0.001**	3.33 (0.92)	2.33 (0.57)	**0.001**

*SD, standard deviation*.

a *Mean reflects the score on a 5-point Likert Scale (1 = strongly disagree; 5 = strongly agree)*.

b *P values are based on Wilcoxon's Signed-Rank Test*.

*Note: The bold values in the tables indicate statistical significance at p < 0.05*.

On the other hand, a number of barriers were perceived to be less important following the intervention with participants recording a lower level of agreement for these barriers 30-days post-intervention. These included the lack of weight management education materials (*P* = 0.001) and clinical practice guidelines (*P* = 0.018), lack of training sessions in weight management (*P* = 0.001) and lack of space for proper counseling (*P* = 0.003). There was also a lower level of agreement on barriers related to the reluctance among patients to obtain advice from pharmacists (*P* = 0.030, pre- vs. 30-day post-intervention) and preference among patients to purchase anti-obesity medications instead of obtaining lifestyle modification advice (*P* = 0.001, pre- vs. 30-day post-intervention).

## Discussion

To the best of the authors' knowledge, this is the first study to evaluate the impact of an educational intervention on the knowledge, attitude, and perceived barriers regarding weight management among Malaysian CPs.

The educational intervention improved the knowledge of CPs regarding various aspects of weight management over time. These findings are in line with the study by Sarayani et al. (6), in which knowledge scores on weight management among Iranian CPs significantly improved following an educational intervention. Interestingly, participants' knowledge scores in the present study were significantly higher after 30 days compared to immediately following the intervention. It should be noted that the participants had a relatively high pre-intervention mean knowledge score (14.5 out of 20); this could explain the minimal improvement immediately after the educational intervention. However, participants could have applied what had been learned during the educational training program on clients seeking weight management advice, and thus further enhanced their knowledge on weight management 30 days after the training program. This is supported by participants' reports of frequent encounters with clients requiring advice regarding weight management issues, where two-third of participants had at least two clients visiting the pharmacy every month to obtain weight-related advice. Indeed, after the intervention, the participants may have a higher appreciation towards the importance of knowledge and skills learned in the training program, and thus expressed significantly higher agreement that only certified pharmacists who have attended weight management training should be eligible to offer WMS. Another possible reason for the observation may lie in the design of the educational training program itself, where training programs with a mixed-method instructional design (lectures in combination with small group training and case discussions) may be effective in improving longer-term knowledge in weight management (7). This was demonstrated in the study by Sarayani et al. (6), in which lectures in combination with small group training resulted in better learning retention over time, compared to didactic lectures or lectures in combination with case discussions.

Within the literature, CPs generally expressed positive views on their role in weight management. They believe that as trained health care professionals, they hold a unique position and therefore have a definite role to play in weight management (8–11). CPs regarded their pharmacies and their WMS offered to be accessible, given that their advice is provided free of charge (8, 9). In this study, after the intervention, the participants had a more positive attitude towards WMS provision in community pharmacy. They had significantly stronger perceptions about the importance of their role to manage overweight and obesity and their professional competence to treat obese patients. It is also encouraging to observe that the participants portrayed more enthusiasm in the provision of WMS after the intervention, in which they perceived significantly more strongly that obesity screening services are important to provide in the pharmacy, and that they should not only provide assistance to overweight/obese patients upon request. These positive attitudes were found to be retained 30 days after the intervention.

CPs have been subjected to scrutiny, where criticism from consumers emerged in the public social media with regards to the perceived conflicts of interest of CPs in selling weight loss products to increase their net revenue (3, 12). The educational intervention may be a good approach to discourage the practices of merely selling weight loss products to manage overweight or obese patients while disregarding non-pharmacological interventions. This was evidenced in our study where the participants significantly more strongly disagreed that they would like to give anti-obesity medications only to achieve weight loss instead of advice on lifestyle/dietary modifications, and that they do not prefer to advise anything on lifestyle/dietary modifications to overweight/obese patients. These beliefs were retained 30 days after the intervention.

Frequently cited barriers in the literature on the provision of community pharmacy-based WMS included a lack of knowledge of obesity and its treatment, a lack of pharmacist time, and a lack of appropriate counseling space (2–5, 8, 9, 13, 14). Educational interventions may help CPs to overcome some of these perceived barriers. For instance, after the intervention, the barrier of not having space in pharmacy to perform proper counseling for weight management and the barrier of not having training sessions in the area of obesity management were perceived to be significantly less important. In contrast, after the intervention, participants perceived significantly more strongly that they do not have manpower at their pharmacy to manage overweight/obese patients. This may be related to the awareness of participants after the educational intervention on various aspects of weight management besides sales of weight loss products, which may require the participants to engage in more personalized and focused patient interactions. In fact, the participants had less strong perceptions of the barrier regarding the tendency of overweight/obese patients to purchase anti-obesity medications instead of getting advice from the pharmacist on lifestyle/dietary modification after the intervention. The situation is probably made worse by difficulties in recruiting and retaining competent auxiliary staff, such as dietitians and nutritionists, to lighten the burden of CPs, especially those who operate the pharmacy independently (9).

CPs frequently cited lack of remuneration or reimbursement to be one of the top barriers to the delivery of WMS (2, 5, 8, 9, 13). In this study, after the intervention, the participants had significantly stronger perceptions about the reluctance of overweight/obese patients to pay pharmacists for WMS as being a barrier, although they were willing to participate in the services even without reimbursement. While the participants expressed significantly higher agreement after the intervention that they should not assist overweight/obese patients only upon being reimbursed, a fee-for-service model would serve to encourage patients' recognition of CPs, who have gone the extra mile to be accredited for the skills of the WMS. This is in line with the findings of a qualitative study among community pharmacists in the United Kingdom of whom most had attended training courses on weight management, where they appeared to be satisfied by their remuneration even though they were not funded for their service, and they expressed their intention to assist more customers by having more advertisements (14).

With the global transition in focus of CPs' roles towards patient-centered services, competency-based weight management training programs may equip pharmacists with professional knowledge and thus facilitate the delivery of WMS. The training intervention in the current study demonstrates that the knowledge and attitude of community pharmacists regarding weight management can be positively improved through a carefully designed training program based on identified needs. In fact, certain barriers to the delivery of WMS were perceived to be less important after the training program. The findings demonstrate the potential effectiveness of brief training interventions for CPs directed towards WMS. In Malaysia, no party has been responsible for providing accredited weight management education for CPs. The findings can serve as a reference for the future planning of such education since key elements of the training program (e.g., dietary and exercise intervention, behavioral modification, pharmacotherapy) are relevant across all community pharmacy settings in Malaysia. It may be pertinent for policymakers to consider a specialized weight management course for CPs that follows the design of the educational training program described in this paper, to provide accreditation and subsequently to reimburse accredited weight management providers among CPs. Importantly, the educational training program should be conducted frequently to ensure sustainability of any positive changes from the intervention. A study among community pharmacists in the United Kingdom has suggested that it would be beneficial if the refresher training on weight management happens on a regular basis (14).

This study had several strengths. This is the first study to evaluate an educational training program to improve weight management knowledge and attitudes among CPs in Malaysia. Another strength of the study was the participation of CPs from both independent and chain pharmacies. However, as the study involved a pilot intervention and was designed to be exploratory, the small sample size may be a limitation that limits the generalizability of the study. In addition, the use of a self-evaluation questionnaire, which depends on accurate and honest reporting from respondents, could affect the responses as it may be subjected to the respondent or recall bias. Furthermore, the use of limited questions may be another limitation of the objective evaluation of knowledge.

## Conclusion

In this study, the educational intervention program improved the knowledge of CPs regarding various aspects of weight management over time. Upon educational intervention, the CPs portrayed more enthusiasm in the provision of WMS in the pharmacy, including obesity screening services. Although certain barriers to WMS were perceived to be less important after the intervention, others such as the lack of reimbursement for services were still perceived to be important, signaling the need for these barriers to be addressed for better implementation of WMS in the community pharmacies across Malaysia.

## Data Availability Statement

The raw data supporting the conclusions of this article will be made available by the authors, without undue reservation.

## Ethics Statement

Ethical approval for this study was obtained from National University of Malaysia, Malaysia (UKMPPI/111/8/JEP-2018-664). The patients/participants provided their written informed consent to participate in this study.

## Author Contributions

RV and WC conceived the study, analyzed the data, and drafted the manuscript. NT and TP assisted with conceptualization of the study, data analysis, and manuscript revision. All authors read and approved the final manuscript.

## Conflict of Interest

The authors declare that the research was conducted in the absence of any commercial or financial relationships that could be construed as a potential conflict of interest.

## Publisher's Note

All claims expressed in this article are solely those of the authors and do not necessarily represent those of their affiliated organizations, or those of the publisher, the editors and the reviewers. Any product that may be evaluated in this article, or claim that may be made by its manufacturer, is not guaranteed or endorsed by the publisher.
